# Nonadherence in Hemodialysis Patients and Related Factors: A Multicenter Study

**DOI:** 10.1097/jnr.0000000000000309

**Published:** 2019-07-16

**Authors:** Nurten Ozen, Fatma Ilknur Cinar, Dilek Askin, Dilek Mut, Turker Turker

**Affiliations:** 1 PhD, RN, Assistant Professor, Department of Nursing, Florence Nightingale Hospital School of Nursing, Demiroglu Bilim University, Istanbul, Turkey; 2 PhD, RN, Associate Professor, Gulhane Faculty of Nursing, University of Health Sciences, Ankara, Turkey; 3 RN, Deparment of Paediatric, Haydarpasa Sultan Abdulhamid Training and Research Hospital, University of Health Sciences, Istanbul, Turkey; 4 RN, Department of Obstetrics and Gynecology, Dogubayazit Doç. Dr. Yasar Eryilmaz State Hospital, Agri, Turkey; 5 MD, Associate Professor, Department of Public Health and Epidemiology, Gulhane School of Medicine, University of Health Sciences, Ankara, Turkey.

**Keywords:** nonadherence, treatment, hemodialysis, dietary and fluid restrictions, medication

## Abstract

**Background:**

Nonadherence to dietary and fluid restrictions, hemodialysis (HD), and medication treatment has been shown to increase the risks of hospitalization and mortality significantly. Sociodemographic and biochemical parameters as well as psychosocial conditions such as depression and anxiety are known to affect nonadherence in HD patients. However, evidence related to the relative importance and actual impact of these factors varies among studies.

**Purpose:**

The aim of this study was to identify the factors that affect nonadherence to dietary and fluid restrictions, HD, and medication treatment.

**Methods:**

This descriptive study was conducted on 274 patients who were being treated at four HD centers in Turkey. The parameters used to determine nonadherence to dialysis treatment were as follows: skipping multiple dialysis sessions during the most recent 1-month period, shortening a dialysis session by more than 10 minutes during the most recent 1-month period, and Kt/V < 1.4. The parameters used to determine nonadherence to dietary and fluid restriction were as follows: serum phosphorus level > 7.5 mg/dl, predialysis serum potassium level > 6.0 mEq/L, and interdialytic weight gain > 5.7% of body weight. The Morisky Green Levine Medication Adherence Scale was performed to determine nonadherence to medication treatment. A patient was classified as nonadherent if he or she did not adhere to one or more of these indices. The Hospital Anxiety and Depression Scale was used to identify patient risk in terms of anxiety and depression. Logistic regression was used to determine the predictors of nonadherence.

**Results:**

The nonadherence rate was 39.1% for dietary and fluid restrictions, 33.6% for HD, and 20.1% for medication. The risk of nonadherence to dietary and fluid restriction was found to be 4.337 times higher in high school graduates (95% CI [1.502, 12.754], *p* = .007). The risk of nonadherence to HD treatment was 2.074 times higher in men (95% CI [1.213, 3.546], *p* = .008) and 2.591 times higher in patients with a central venous catheter (95% CI [1.171, 5.733], *p* = .019). Longer duration in HD resulted in 0.992 times decrease in risk of nonadherence to treatment (95% CI [0.986, 0.998], *p* = .005).

**Conclusions/Implications for Practice:**

Educational status, being male, having a central venous catheter, and having a short HD duration were found to be risk factors for nonadherence. Nurses must consider the patient's adherence to the dietary and fluid restrictions, HD, and medication treatment at each visit.

## Introduction

Hemodialysis (HD) is a treatment method that requires adherence to prescribed medication, dialysis treatment, and dietary and fluid restrictions to ensure success (Denhaerynck et al., 2007). Adherence is defined as “the extent to which a person's behavior corresponds with the agreed recommendations of a healthcare provider in terms of taking medications, following a recommended diet and/or executing lifestyle changes” (Sabaté, 2003). Nonadherence to dialysis treatment results in undesirable consequences such as bone demineralization, pulmonary edema, and metabolic disorders and leads to the development of cardiovascular disorders and, finally, death (Denhaerynck et al., 2007). Nonadherence to dietary and fluid restrictions and medication treatment were found in the Dialysis Outcomes and Practice Patterns Study (Saran et al., 2003) to increase the risks of hospitalization and mortality significantly.

Nonadherence to dialysis treatment has been generally reported at rates between 8.5% and 22.1% worldwide and, in one study, as high as 86% (Matteson & Russell, 2010). Failure to attend all dialysis sessions, which is an important indicator of adherence to dialysis treatment, has also been noted at rates of 7%–32% (Durose, Holdsworth, Watson, & Przygrodzka, 2004; Saran et al., 2003). In HD patients, nonadherence to fluid restrictions has been reported as 9.7%–75.3% (Kugler, Maeding, & Russell, 2011); nonadherence to dietary restrictions, as 2%–80.4% (Kugler et al., 2011; Saran et al., 2003); and nonadherence to medication treatment, as 15.4%–99% (Saran et al., 2003).

HD patients should take responsibility for many aspects of their treatment to successfully manage this chronic condition. These aspects include dietary and fluid restriction adherence, medication adherence, and attending all HD sessions (Kammerer, Garry, Hartigan, Carter, & Erlich, 2007). Dietary and fluid restriction adherence is crucial for treatments to be successful, and failure may lead to increased rates of complications (and related costs) and decreased survival (Unruh et al., 2005). Study findings have shown associations between nonadherence and sociodemographic factors such as age, gender, and educational level as well as social support status, dialysis duration, vascular access used, anxiety, depression, smoking, and alcohol use with the nonadherence (Akman et al., 2007; Clark, Farrington, & Chilcot, 2014; Higgins, 2006; Jin, Sklar, Min Sen Oh, & Chuen Li, 2008; Russell et al., 2011).

Understanding the factors that may influence treatment outcomes in patients on HD is important for the delivery of optimum healthcare. However, the number of studies that have investigated the effects of patient sociodemographic and psychosocial statuses on adherence is inadequate (Karamanidou, Clatworthy, Weinman, & Horne, 2008; Saran et al., 2003; Unruh et al., 2005). Furthermore, the effects of anxiety on HD patients are unknown (Feroze, Martin, Reina-Patton, Kalantar-Zadeh, & Kopple, 2010), and no study has investigated the relationship between anxiety and adherence in HD patients (Mellon, Regan, & Curtis, 2013).

Thus, the purposes of this study were (a) to evaluate the prevalence of patient nonadherence in terms of dietary and fluid restrictions, HD, and medication treatment and (b) to identify the factors that influence nonadherence in patients undergoing HD.

## Methods

### Design and Sample

This descriptive study was conducted between November 2015 and June 2016 at four dialysis centers in Ankara, the capital of Turkey. Inclusion criteria were as follows: (a) patients who had received HD treatment for 4 hours a day, 3 days a week for a minimum of 6 months; (b) age of 18 years or older; (c) no communication problems; and (d) no Alzheimer disease or any psychiatric problems related to cognitive disorders such as psychosis. The study was completed with 274 patients who met the inclusion criteria and agreed to participate.

### Data Collection

Data collection forms were completed by the investigators based on face-to-face interviews given during the second hour of HD treatment. Three investigators collected the data. One had a dialysis nurse certificate, and the remaining two had worked in a dialysis unit for 1 year. The medical data were recorded by the investigators based on patient medical charts. The patients were asked to think about the most recent month when answering the questions. Completion of the form took about 15–20 minutes.

### Ethical Consideration

Permission was obtained from the hospital ethics committee (Gulhane Military Medical Academy Ethical Committee, Session Number: 12, Registration Number: 383) and the dialysis centers where the study was conducted. After the purpose of the study was explained to the patients by the investigators, written consent was obtained from those who agreed to participate.

### Measures

#### Sociodemographic and clinical details

The sociodemographic and clinical information form was developed by the investigators after reviewing the relevant literature (Denhaerynck et al., 2007; Kim, Evangelista, Phillips, Pavlish, & Kopple, 2010; Kugler et al., 2011; Matteson & Russell, 2010). Information on age, gender, marital status, employment status, income category, education, smoking status, type of vascular access used, duration of dialysis, cause of chronic kidney failure, and comorbidity status were gathered using this form.

### Psychosocial Measures

#### Hospital Anxiety and Depression Scale

This scale was developed by Zigmond and Snaith (1983) to identify risks of anxiety and depression in patients and also to measure changes in level and severity of these risks. The Hospital Anxiety and Depression Scale (HADS) is widely used for initial assessments of depression and/or anxiety disorders and is commonly used with HD patients (Cukor et al., 2008; Mellon et al., 2013; Sharp, Wild, Gumley, & Deighan, 2005). The validity and reliability of the scale were tested in Turkey by Aydemir, Güvenir, Küey, and Kültür (1997). The scale is used to diagnose anxiety and depression quickly and to determine risk groups. It is not intended for use as a tool to diagnose patients with physical diseases. Half (7) of the 14 questions measure anxiety, and the other half (7) measure depression. The responses are scored from 0 to 3 using a 4-point Likert scale. The total possible range of scores for this scale is 0–21. The cutoff points for the Turkish version of HADS were found to be 10 for the anxiety subscale and 7 for the depression subscale (Aydemir et al., 1997). The reliability of the HADS was .64 in this study.

#### Multidimensional Scale of Perceived Social Support

The Multidimensional Scale of Perceived Social Support (MSPSS), developed by Zimet, Dahlem, Zimet, and Farley (1988), is commonly used with HD patients (Ahrari, Moshki, & Bahrami, 2014; Fincham, Kagee, & Moosa, 2008). The validity and reliability study for this scale was performed in Turkey by Eker and Arkar in 1995. The MSPSS form was reviewed in terms of factor structure, validity, and reliability by Eker, Arkar, and Yaldiz in 2001. The 12 items of the scale are all scored using a 7-point Likert-type scale ranging from “definitely no” to “definitely yes.” There are three subgroups, consisting of family, friends, and private support, that reflect the support sources and contain four items each. The total possible range of scores for each subscale is 4–28, and for the MSPSS, it is 12–84. The scale has no cutoff point, and high scores indicate a high level of perceived social support (Eker et al., 2001). The reliability for the MSPSS was .95 in this study.

### Nonadherence Measures

Nonadherence was evaluated using parameters that were used in the guide published by the National Kidney Foundation (Hemodialysis Adequacy 2006 Work Group, 2006) and in related studies (Kugler et al., 2011; Matteson & Russell, 2010; Mellon et al., 2013; Saran et al., 2003).

HD treatment nonadherence was defined in this study as (a) skipping more than one dialysis session during the most recent 1-month period, (b) shortening a dialysis session by > 10 minutes during the most recent 1-month period, and (c) Kt/V < 1.4. Nonadherence to dietary and fluid restrictions was defined as (a) serum phosphorus level > 7.5 mg/dl, (b) predialysis serum potassium level > 6.0 mEq/L, and (c) interdialytic weight gain (IDWG) > 5.7% of body weight. Medication nonadherence was defined using the Morisky Green Levine Medication Adherence Scale. A patient was classified as nonadherent if he or she did not adhere to one or more of these indices (Mellon et al., 2013).

IDWG was defined as the difference between predialytic weight and the weight at the end of the previous dialysis session, as averaged over 12 HD sessions (Mellon et al., 2013). If a patient was unable to attend a session because of hospitalization, this was not counted as nonadherence. The Daugirdas formula was used to calculate the Kt/V value (Daugirdas, 1993). Serum potassium, phosphorus, and Kt/V values were obtained by taking the average of the patient values over the last 3 months.

#### Morisky Green Levine Medication Adherence Scale

The previously validated, four-item Morisky Green Levine Medication Adherence Scale was used to assess self-reported medication adherence (Morisky, Green, & Levine, 1986). Each item in this scale queries the patient on whether a specific nonadherence behavior has taken place. Each of the items is answered with either “yes” (1) or “no” (0). The total possible range of scores for this scale is 0–4, with a higher score indicating a higher degree of self-reported nonadherence. In this study, patients with a score of 0 were categorized as “adherent,” whereas all others were categorized as “nonadherent,” following the example of Khanderia et al. (2008). The reliability of the Morisky Green Levine Medication Adherence Scale was .796 in this study.

### Data Analysis

SPSS for Windows Version 15.0 (SPSS Inc., Chicago, IL, USA) was used to evaluate the data and to conduct statistical analyses. Descriptive statistics were presented in terms of numbers and percentages for discrete variables (such as gender and marital status) and as mean ± standard deviation for continuous variables (such as age, calcium, and albumin value). The compliance of the data with a normal distribution was evaluated using the Kolmogorov–Smirnov test. The Mann–Whitney *U* test was used for intergroup comparisons to identify nonnormal distributions. The chi-square test was used for nominal data in pairwise comparisons. Multivariable logistic regression analysis was used to determine the factors that influenced nonadherence, with variables having a *p* value of .25 or less in the single comparisons included in the regression analysis as candidates. In addition, clinically significant variables (marital, income, and smoking status; use of erythropoietin; and IDWG) were included in the regression analysis. A *p* value < .05 was accepted as an indicator of a significant difference in statistical decisions.

## Results

### Patient Characteristics

The descriptive characteristics of our patients are presented in Table 1. The mean age was 62.57 years (*SD* = 13.24 years); 54.4% were female, 52.6% were primary school graduates, and 79.9% were married. The cause of chronic kidney failure was hypertension in 25.2%, and the mean HD treatment duration was 67.54 months (*SD* = 54.98 months). The anxiety risk was low in 89.4%, and the depression risk was high in 80.7%. The total social support score was 52.78 (*SD* = 20.71).

**TABLE 1.**
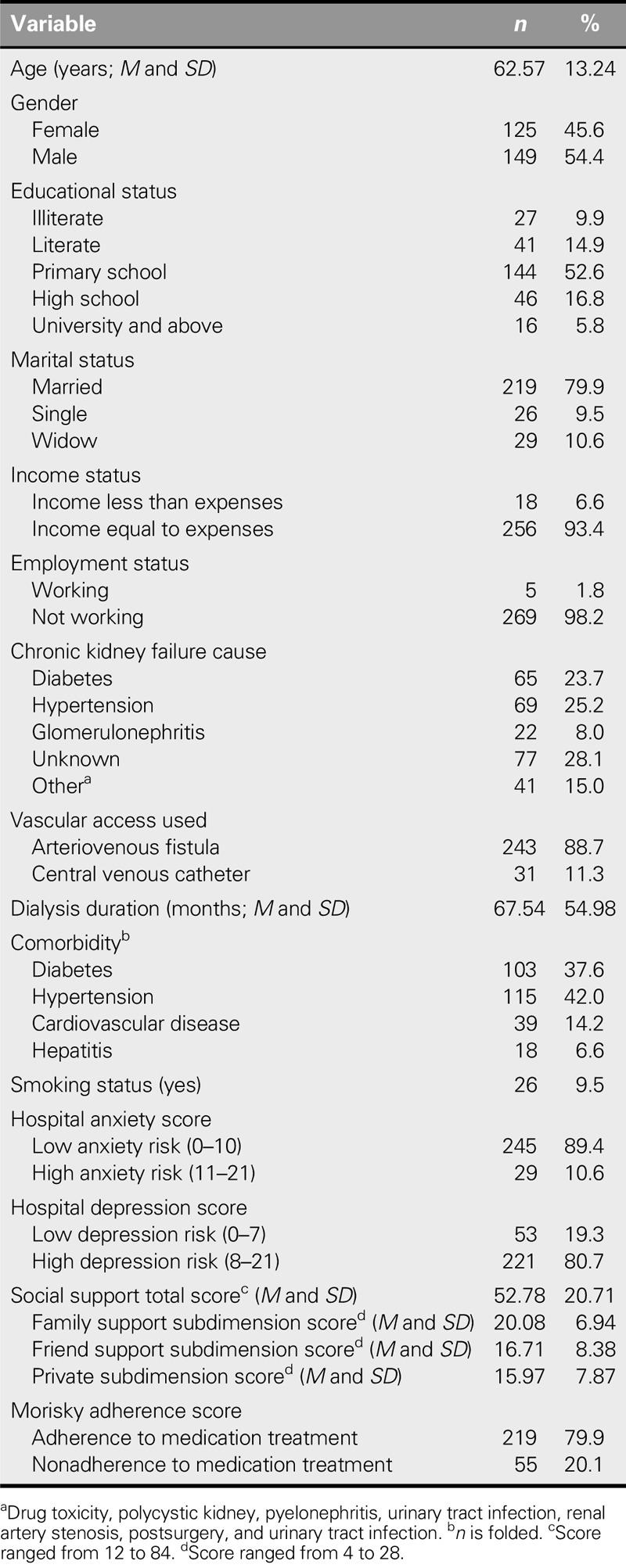
Descriptive Characteristics of the Patients (*N* = 274)

### Nonadherence Measures

The distribution of the parameters used in the evaluation of nonadherence is presented in Table 2. IDWG was > 5.7% of dry weight in 6.2% of the patients, serum PO_4_ was > 7.5 mg/dl in 27.4%, and predialysis serum potassium was > 6.0 mEq/L in 15.0%. Kt/V was < 1.4 in 29.9% of the patients.

**TABLE 2.**
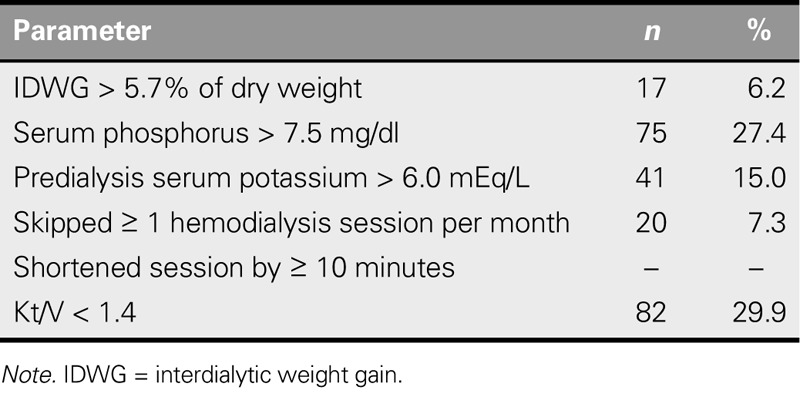
Distribution of the Parameters Used to Evaluate Nonadherence *(N = 274)*

The comparison of the patients' nonadherence to dietary and fluid restrictions, HD, and medication treatment are presented in Table 3 together with their sociodemographic and medical characteristics. The nonadherence rate was 39.1% for dietary and fluid restrictions, 33.6% for HD, and 20.1% for medication.

**TABLE 3.**
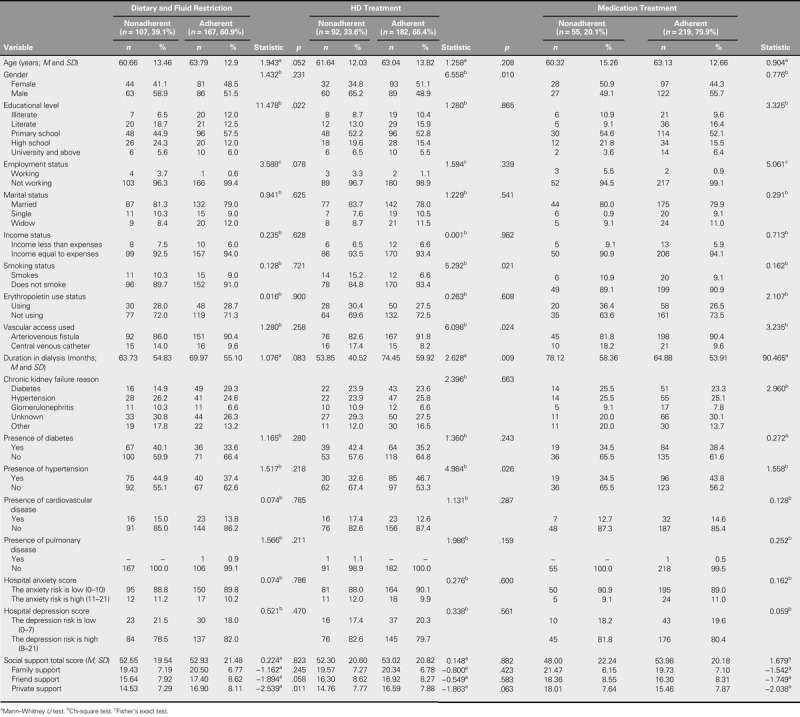
Relationship Between Nonadherence to Dietary and Fluid Restrictions, HD, and Medication Treatment and the Sociodemographic and Medical Characteristics of the Patients (*N* = 274)

Primary school graduates were found to be statistically significantly more adherent to their dietary and fluid restrictions (χ^2^ = 11.478, *p* = .022). Being male (χ^2^ = 6.558, *p* = .010) and not having hypertension (χ^2^ = 4.984, *p* = .026) were more common in the group nonadherent to HD treatment. The percentage of patients who did not smoke (χ^2^ = 5.292, *p* = .021), the percentage with arteriovenous fistula (χ^2^ = 6.098, *p* = .024), and the dialysis duration (*Z* = 2.628, *p* = .009) were found to be statistically significantly higher in the group adherent to HD treatment. The employed patient rate was statistically significantly higher in the adherent patient group (χ^2^ = 5.061, *p* = .024). The dialysis duration was also statistically significantly higher in the patients who were nonadherent to their medication compared with those who were adherent (*Z* = 90.465, *p* = .050).

### Regression Analysis

The results of the logistic regression analysis performed to identify the factors effective on nonadherence of the patients with dietary and fluid restrictions, HD, and medication treatment are shown in Table 4. The risk of nonadherence to dietary and fluid restrictions was found to be 4.337 times higher in high school graduates (95% CI [1.502, 12.754], *p* = .007). The risk of nonadherence to HD treatment was found to be 2.074 times higher in men (95% CI [1.213, 3.546], *p* = .008). The risk of nonadherence to HD treatment was found to be 2.591 times higher in patients with a central venous catheter (CVC; 95% CI [1.171, 5.733], *p* = .019). A longer duration in HD resulted in 0.992 times lower risk of nonadherence to treatment (95% CI [0.986, 0.998], *p* = .005).

**TABLE 4.**
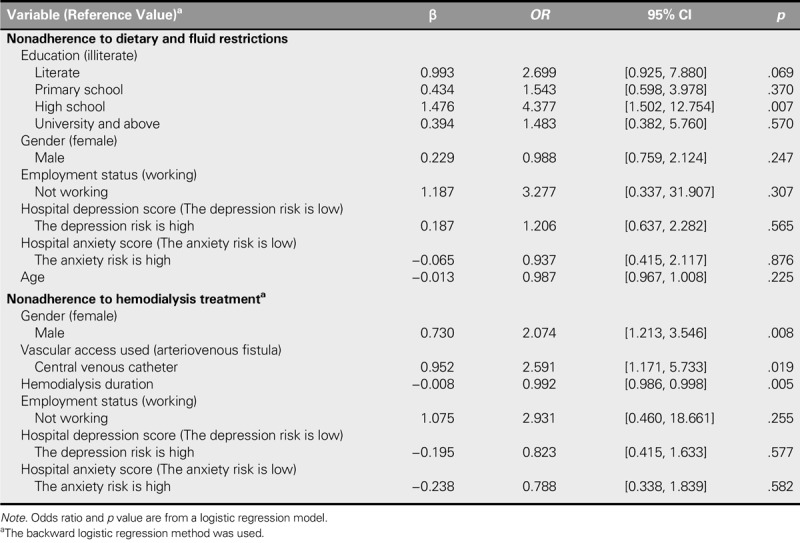
The Logistic Regression Evaluation of the Factors Affecting Nonadherence (*N* = 274)

## Discussion

This study aimed to identify the risk factors that lead to nonadherence to dietary and fluid restrictions, HD, and medication treatment. The nonadherence rate was found to be 39.1% for dietary and fluid restrictions, 33.6% for HD, and 20.1% for medication. Educational status, being male, having a CVC, and having a short HD duration were identified as significant risk factors for nonadherence.

In this study, the risk of nonadherence to dietary and fluid restrictions was found to be 4.337 times higher in high school graduates than in illiterate subjects. The effect of educational level on nonadherence to dietary and fluid restrictions in HD patients is not clear. Some studies have identified low level of education as a risk factor for nonadherence (Rambod, Peyravi, Shokrpour, & Taghi Sareban, 2010; Safdar, Baakza, Kumar, & Naqvi, 1995), although Chan, Zalilah, and Hii (2012) reported no significant relationship. Some studies have suggested that level of education affects adherence but that understanding treatment instructions and the importance of treatment is probably relatively more important (Krueger, Berger, & Felkey, 2005). Research shows that higher levels of knowledge do not necessarily increase patient adherence (Morgan, 2000). It may be difficult for highly educated patients to adhere because of professional/social obligations and status. These factors require more extensive research using quantitative studies.

This study found that being male is a risk factor for nonadherence to HD treatment. Besides, nonadherence to the treatment decreased as HD duration increased. No significant relationship was found between HD duration and either gender or nonadherence to the treatment in Ibrahim, Hossam, and Belal (2015). Saran et al. (2003) found higher rates of nonadherence to HD treatment in men, whereas Wells (2015) reported that many men thought that they had lost their role because of cultural reasons and that these roles were fulfilled by other family members. Thus, understanding the opinions of male patients about this issue should be prioritized by healthcare staff, and appropriate support should be provided. The reason for the high incidence of nonadherence in men compared with women may be cultural. Many men may think that they are unable fulfill their household duties when they receive HD treatment for 4 hours a day, 3 days a week, and experience fatigue and drowsiness after dialysis. This loss of autonomy may encourage men to stop perceiving themselves as the “man of the house,” which is an important role in the Turkish cultural context.

This study found having a CVC to be a risk factor for nonadherence to HD treatment. The pain and sense of discomfort experienced during the intervention for arteriovenous fistula were reported to be one of the factors making it difficult to adhere to dialysis among the patients in Madeiro, Machado, Bonfim, Braqueais, and Lima (2010). Prior studies have not investigated whether vascular access is a risk factor for nonadherence in HD treatment (Ibrahim et al., 2015; Saran et al., 2003). CVCs used in dialysis treatment cause repeated hospitalizations due to the high risk of infection and thrombosis. Furthermore, they reduce the comfort of patients, cause visual disturbance, and limit mobility (Frykholm et al., 2014). CVCs were found to be a risk factor in our study as well, possibly because of these reasons. Therefore, new studies on the issue are required.

This study found that longer HD duration was associated with a reduced risk of nonadherence to treatment. The reason may be that patients evaluate the effects of dialysis on their body and learn to cope with complications by talking to other patients and the healthcare staff. A longer treatment period typically leads to greater interaction (Chan et al., 2012; McDonald, Garg, & Haynes, 2002). In Allen, Wainwright, and Hutchinson (2011), many patients reported that they received information about the management of their disease through observation during HD treatment and by talking to healthcare staff and other patients. Patients frequently perceive that knowing more about their disorder gives them greater autonomy. Therefore, they regularly try to understand the details of their disease, its treatment, the related medical system, and the unique ways in which their body responds to the treatment.

One limitation of this study is that the sample population was affected by a number of other serious, comorbid illnesses such as diabetes, hypertension, and cardiovascular disease. These comorbidities may have affected biochemical levels in ways that were not controlled for in this study because cross-sectional studies are not able to explore changes in nonadherence behaviors.

### Conclusions/Implications for Practice

Educational status, being male, having a CVC, and having a short HD duration were identified as risk factors for nonadherence. These risk factors must be taken into account when planning HD treatment for patients. In developing strategies to improve adherence in HD units, nurses should look for biochemical and behavioral markers of nonadherence, including missed treatments and excess IDWG, among others. Nurses must consider patient adherence to dietary and fluid restrictions, HD, and medication treatment at each visit. One of the main factors in nonadherence is patients not attending dialysis treatment, with possible reasons including transportation problems, forgetting the appointment, and so on. Moreover, adherence patterns may change over time. Therefore, it is essential to collect data regularly on factors that affect nonadherence. In addition, nurses should develop strong support relationships with the patient, identify barriers, and offer strategies to help patients improve adherence. Nursing care plans should be individualized and used in the standard care provided in HD units. The relationship between nonadherence and the related factors should be investigated on larger populations in future studies.
